# Superior accuracy of mid-regional proadrenomedullin for mortality prediction in sepsis with varying levels of illness severity

**DOI:** 10.1186/s13613-017-0238-9

**Published:** 2017-02-10

**Authors:** David Andaluz-Ojeda, H. Bryant Nguyen, Nicolas Meunier-Beillard, Ramón Cicuéndez, Jean-Pierre Quenot, Dolores Calvo, Auguste Dargent, Esther Zarca, Cristina Andrés, Leonor Nogales, Jose María Eiros, Eduardo Tamayo, Francisco Gandía, Jesús F. Bermejo-Martín, Pierre Emmanuel Charles

**Affiliations:** 1 Servicio de Medicina Intensiva, Hospital Clínico Universitario, Avda Ramón y Cajal 3, 47005 Valladolid, Spain; 2 Group for Biomedical Research in Sepsis (Bio∙Sepsis), Hospital Clínico Universitario, Avda Ramón y Cajal 3, 47005 Valladolid, Spain; 3 Division of Pulmonary and Critical Care Medicine, Department of Medicine, Loma Linda University, Loma Linda, CA USA; 4 Service de Réanimation Médicale, Hôpital Bocage Central, C.H.U. DIJON, 14 rue Gaffarel, B.P. 77908-21079, Dijon Cedex, France; 5 Servicio de Análisis Clínicos, Hospital Clínico Universitario, Avda Ramón y Cajal 3, 47005 Valladolid, Spain; 6 Departmento de Microbiología, Facultad de Medicina, Universidad de Valladolid, Avda/Ramón y Cajal 7, 47005 Valladolid, Spain; 7 Servicio de Anestesiología y Reanimación, Hospital Clínico Universitario, Avda/Ramón y Cajal 3, 47005 Valladolid, Spain

**Keywords:** MR-proADM, Biomarkers, Sepsis, Mortality, SOFA

## Abstract

**Background:**

The use of novel sepsis biomarkers has increased in recent years. However, their prognostic value with respect to illness severity has not been explored. In this work, we examined the ability of mid-regional proadrenomedullin (MR-proADM) in predicting mortality in sepsis patients with different degrees of organ failure, compared to that of procalcitonin, C-reactive protein and lactate.

**Methods:**

This was a two-centre prospective observational cohort, enrolling severe sepsis or septic shock patients admitted to the ICU. Plasma biomarkers were measured during the first 12 h of admission. The association between biomarkers and 28-day mortality was assessed by Cox regression analysis and Kaplan–Meier curves. Patients were divided into three groups as evaluated by the Sequential Organ Failure Assessment (SOFA) score. The accuracy of the biomarkers for mortality was determined by area under the receiver operating characteristic curve (AUROC) analysis.

**Results:**

A total of 326 patients with severe sepsis (21.7%) or septic shock (79.3%) were enrolled with a 28-day mortality rate of 31.0%. Only MR-proADM and lactate were associated with mortality in the multivariate analysis: hazard ratio 8.5 versus 3.4 (*p* < 0.001). MR-proADM showed the best AUROC for mortality prediction at 28 days in the analysis over the entire cohort (AUROC [95% CI] 0.79 [0.74–0.84]) (*p* < 0.001). When patients were stratified by the degree of organ failure, MR-proADM was the only biomarker to predict mortality in all severity groups (SOFA ≤ 6, SOFA = 7–12, and SOFA ≥ 13), AUROC [95% CI] of 0.75 [0.61–0.88], 0.74 [0.66–0.83] and 0.73 [0.59–0.86], respectively (*p* < 0.05). All patients with MR-proADM concentrations ≤0.88 nmol/L survived up to 28 days. In patients with SOFA ≤ 6, the addition of MR-proADM to the SOFA score increased the ability of SOFA to identify non-survivors, AUROC [95% CI] 0.70 [0.58–0.82] and 0.77 [0.66–0.88], respectively (*p* < 0.05 for both).

**Conclusions:**

The performance of prognostic biomarkers in sepsis is highly influenced by disease severity. MR-proADM accuracy to predict mortality is not affected by the degree of organ failure. Thus, it is a good candidate in the early identification of sepsis patients with moderate disease severity but at risk of mortality.

**Electronic supplementary material:**

The online version of this article (doi:10.1186/s13613-017-0238-9) contains supplementary material, which is available to authorized users.

## Background

Sepsis remains the primary cause of death in intensive care unit (ICU) patients despite improvements in antibiotic and early hemodynamic management. In Europe, sepsis occurrence in acutely ill patients results in an ICU mortality rate ranging between 27 and 54% depending on the severity [1]. In the USA, the Centre for Disease Control estimates that 500,000 people develop sepsis and 200,000 die each year [2, 3]. The prompt diagnosis and assessment of high risk sepsis patients is therefore highly desirable, increasing the possibility of initiating early and specific treatments. Thus, clinical severity scores such as Sequential Organ Failure Assessment (SOFA) score can play a critical role [4]. However, the isolated use of these scoring systems to guide decision-making in sepsis has been heavily criticized [5]. A standardized assessment tool for the early identification of sepsis patients upon admission with a range of severity levels would be of dramatic value in aiding clinical decision-making and optimizing the use of health care resources. Accordingly, a number of prognostic biomarkers have been proposed in the field of sepsis over the last decades—many more than in other diseases. Most of these molecules are hormones, cytokines or circulating proteins related to inflammation or the coagulation system and may require considerable time, effort and costs to be measured [6].

Adrenomedullin (ADM) is a peptide which can act as a hormone and is produced by multiple tissues during physiologic and infectious stress with varying physiological functions, including vasodilatory, anti-inflammatory and antimicrobial activity, which is further enhanced by its regulation and modulation of complement activity [7]. Thus, ADM is considered a “hormokine”, characterized by a hormone-like behaviour in non-inflammatory conditions when it is only produced by endocrine cells, and by a cytokine-like behaviour in sepsis when it is ubiquitously hyper-expressed. Moreover, exogenous ADM has been shown to reduce acute lung injury, vascular permeability and death in animal models of sepsis, whilst endogenous over-expression similarly ameliorates the sepsis insult [8, 9]. Measurement of circulating ADM is complicated by a rapid degradation and clearance from the circulation, and is further masked by a binding protein (complement factor H), preventing its detection by standard immunoassay. The mid-regional fragment of proadrenomedullin (MR-proADM), comprising of amino acids 45–92, is more stable and directly reflects levels of the rapidly degraded active ADM peptide [10]. Increased MR-proADM concentrations have been identified in the plasma of patients with community acquired pneumonia (CAP) and are widely used in the risk and severity assessment of this condition [11–13]. However, very few are available for severe sepsis and septic shock patients. Additionally, the influence of disease severity on the performance of prognostic biomarkers in sepsis has not been appropriately studied yet.

In this study, we aimed to evaluate the ability of MR-proADM levels to predict 28-day mortality in sepsis patients, compared to other standard biomarkers (procalcitonin (PCT), C-reactive protein (CRP), and lactate), in three different levels of disease severity as measured by the SOFA score.

## Methods

### Patient selection, inclusion and exclusion criteria

This study was a prospective observational cohort of patients recruited consecutively from two intensive care units (ICU) in Spain and France. Adult patients with age ≥18 years and admitted to the ICU from April 2013 to January 2016 were enrolled within 12 h after meeting criteria for severe sepsis or septic shock, based on the SEPSIS-2 definition by the American College of Chest Physicians/Society of Critical Care Medicine Consensus Conference [14]. Enrolled patients also had SOFA score ≥2 and therefore met criteria for the new SEPSIS-3 definition for sepsis [15]. Patients with human immunodeficiency virus (HIV) infection and those undergoing radiotherapy or receiving immunosuppressive drugs, including chemotherapy or systemic steroids, in the 3 months prior to admission to the ICU were considered to be immunosuppressed. Exclusion criteria were age <18 years, the presence of pregnancy, the absence of a blood sample available for biomarker profiling within the first 12 h following ICU admission, or lack of informed consent. Clinical data recorded from the medical records included demographics, comorbidities, laboratories, microbiology, and biomarker levels. The severity of illness was assessed on admission by calculating the Sequential Organ Failure Assessment (SOFA) score.

### Biomarker evaluation

Plasma samples for biomarker profiling were collected as close as possible to the moment of ICU admission, and always within the first 12 h. Plasma MR-proADM measurement was performed by TRACE technology (Time Resolved Amplified Cryptate Emission) using a new sandwich immunoassay (Kryptor Compact Plus Analyser, BRAHMS, Hennigsdorf, Germany); limit of detection 0.05 nmol/L. PCT measurement was performed by electrochemiluminescence immunoassay (ECLIA) on a chemistry analyser (Cobas 6000, Roche Diagnostics, Meylan, France); limit of detection 0.02 ng/ml. Serum CRP and lactate were measured by particle-enhanced immunoturbidimetric and colorimetric assay, respectively (e501 Module Analyser, Roche Diagnostics, Meylan, France); limit of detection 0.15 mg/dL and 0.2 mmol/L, respectively.

### Statistical analysis

Differences in demographic and clinical characteristics between survivors and non-survivors were assessed using the Chi-square test for categorical variables. Student’s *t* test or Mann–Whitney *U* test were, respectively, used to compare continuous variables based upon the presence or absence of normal distribution. The association between biomarkers and the risk of mortality was assessed by Cox regression analysis, adjusted by confounding variables. Time was censored at 28 days following admission to the ICU. The first 24 h of ICU admission was considered as day 1 in the analysis. Variables yielding a *p* < 0.05 in the univariate regression analysis were further included in the multivariate analysis. Biomarkers were log transformed in order to reach a normal distribution. The impact of biomarkers on mean survival time was assessed by using Kaplan–Meier curves and the Mantel–Haenszel log-rank test. Similar to the Cox regression analysis, time was censored at 28 days following admission to the ICU. Accuracy and predictive values of the biomarkers for mortality were evaluated by calculating the area under the receiver operating characteristic (AUROC) curve. Patients were distributed into three groups depending on disease severity as assessed by the SOFA score using two predefined cut-offs, one with a sensitivity close to 90% and the other showing a specificity close to 90% for detecting non-survivors at 28 days (Additional file 1: Figure S1). Data were analysed by using the IBM SPSS 20.0 software (SPSS, Chicago, Ill).

## Results

### Patient characteristics and biomarker concentrations

Three hundred and twenty-six patients (326) with severe sepsis (21.7%) or septic shock (79.3%) were enrolled with a 28-day mortality rate of 25.5 and 34.9% in Valladolid and Dijon, respectively, and an overall mortality rate of 31.0% across both sites (Table 1). The median age was 65 years and 54.3% of patients were male. Compared to survivors, non-survivors were older and presented with higher SOFA scores, and an increased incidence of septic shock, mechanical ventilation, renal replacement therapy, neoplasia, cardiovascular disease, chronic renal failure, immunosuppression, and respiratory disease (all *p* < 0.05). The most common source of infection was of respiratory and urologic origin, regardless of outcome. Mortality rates depending on the source of infection were as follows: 37.2% in patients suffering from a respiratory infection, 32.4% in those with an urological infection, 28.6% in patients with an abdominal infection, 35.5% in those showing a primary or secondary bacteremia and 25.6% in those patients with an infection of other origin. Regarding microbiological identification, both survivors and non-survivors showed a similar presence of Gram−, Gram+ and virus pathogens. Fungal infections were more frequent in non-survivors. The most common cause of death was multi-organ dysfunction syndrome (*n* = 58; 57.4%), followed by refractory shock (*n* = 9; 8.9%) and refractory hypoxemia (*n* = 8; 7.9%). A limitation of therapeutic effort was applied to 21 patients. MR-proADM, PCT and lactate concentrations were all significantly elevated in non-surviving patients compared to survivors (all *p* < 0.01), whereas CRP levels remained similar in both groups. Levels of MR-proADM depending on the source of infection were as follows [median (interquartile range)] respiratory infection [3.6 nmol/L (5.6)], urological infection [4.6 nmol/L (5.4)], abdominal infection [4.9 nmol/L (6.5)], bacteremia [3.8 nmol/L (5.1)], and [3.5 nmol/L (5.8)] in infections of other origin. Levels of MR-proADM depending on the infecting microbe were [median (interquartile range)]: fungal infection [6.1 nmol/L (5.6)], Gram − bacteria [4.9 nmol/L (5.9)], Gram + bacteria [4.1 nmol/L (6.2)] or viruses [1.2 nmol/L (3.4)].

**Table 1:** Clinical characteristics of the patients: data are presented as mean (S.D.) or median (IQR) where appropriate

	Survivors *n* = 225	Non-survivors *n* = 101	Total *n* = 326	*p*
Patients from Valladolid (*n*, %)	102 (45.3%)	35 (34.7%)	137	0.071
Patients from Dijon (*n*, %)	123 (54.7%)	66 (65.3%)	189	
Male (*n*, %)	133 (59.1%)	68 (67.3%)	201 (61.4%)	0.098
Age (years) (mean, SD)	63 (14)	69 (12)	65.4 (14)	<0.001
SOFA (mean, SD)	8 (3.4)	11 (3.5)	9 (3.7)	<0.001
Septic shock (*n*, %)	152 (67.5%)	87 (86.1%)	239 (73.3%)	0.020
Mechanical ventilation (*n*, %)	150 (66.7%)	89 (88.1%)	239 (73.3%)	<0.001
RRT (*n*, %)	40 (17.7%)	45 (44.6%)	85 (26.2%)	<0.001
ICU stay (days) (mean, SD)	12.9 (18)	7.7 (6.7)	11.2 (15.6)	0.012
Neoplasia (*n*, %)	47 (21%)	35 (34.7%)	82 (25.2%)	0.007
Diabetes (*n*, %)	58 (25.8%)	29 (28.7%)	87 (26.7%)	0.330
COPD (*n*, %)	33 (14.7%)	16 (15.8%)	49 (15%)	0.450
Cardiovascular disease (*n*, %)	56 (25%)	41 (40.6%)	97 (29.8%)	0.030
Chronic renal failure (*n*, %)	16 (7.1%)	16 (15.8%)	32 (9.8%)	0.014
Immunosuppression (*n*, %)	21 (9.3%)	25 (24.8%)	46 (14.1%)	<0.001
Respiratory infection (*n*, %)	98 (43.6%)	58 (57.4%)	156 (48%)	0.014
Urologic infection (*n*, %)	75 (33.3%)	36 (35.6%)	111 (34%)	0.380
Abdominal infection (*n*, %)	25 (11.1%)	10 (9.9%)	35 (10.7%)	0.450
Other infection (*n*, %)	32 (14%)	11 (10.9%)	43 (13%)	0.40
Primary or secondary bacteremia (*n*, %)	69 (30.7%)	38 (37.6%)	107 (32.8%)	0.130
Gram − bacteria (*n*, %)	62 (27.6%)	28 (27.7%)	90 (27.6%)	0.975
Gram + bacteria (*n*, %)	47 (20.9%)	22 (21.8%)	69 (21.2%)	0.855
Fungi (*n*, %)	3 (1.3%)	5 (5%)	8 (2.5%)	0.050
Virus (*n*, %)	15 (6.7%)	5 (5%)	20 (6.1%)	0.550
MR-proADM (nmol/L) (median, IQR)	2.68 (3.56)	7.44 (6.84)	3.62 (5.6)	<0.001
Lactate (mmol/L) (median, IQR)	2.00 (1.54)	3.60 (5.53)	2.12 (2.28)	<0.001
CRP (mg/dl) (median, IQR)	147.8 (193.6)	163.0 (181.9)	155.0 (189)	0.200
PCT (ng/ml) (median, IQR)	2.9 (17.5)	5.8 (36.7)	3.54 (27.5)	0.001

Values expressed in percentages (%) indicate the proportion of survivors and non-survivors at 28 days for specific variables

### Survival analysis

**Fig. 1:**
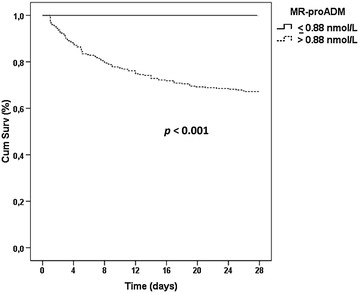
Kaplan–Meier analysis for mortality prediction at 28 days

**Fig. 2:**
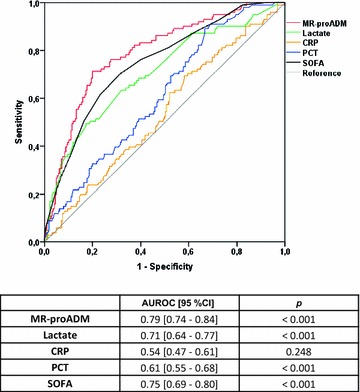
AUROC analysis for identifying non-survivors at 28 days (entire cohort)

MR-proADM, PCT and lactate showed a significant association with mortality in the univariate Cox regression analysis (Table 2). After adjusting for confounders and compared to PCT, CRP, and lactate, MR-proADM showed the strongest independent association with the risk of mortality (hazard ratio 8.5; 95% confidence interval 4.2–17.4; *p* < 0.001; Table 2). In addition, Kaplan–Meier analysis showed that no patients with a MR-proADM value ≤0.88 nmol/L died in the first 28 days following ICU admission (Fig. 1). This cut-off was selected since it provided a sensitivity of 100% in identifying non-survivors in the AUROC (Fig. 2).

**Table 2:** Uni- and multivariate Cox regression analysis for mortality prediction at 28 days following ICU admission

	Univariate		Multivariate	
	HR (95% CI)	*p*	HR (95% CI)	*p*
MR-proADM	11.2 (6.3–19.8)	<0.001	8.5 (4.2–17.4)	<0.001
Lactate	3.8 (2.6–5.5)	<0.001	3.4 (2.0–5.8)	<0.001
CRP	1.3 (0.8–1.9)	0.266	–	–
PCT	1.4 (1.2–1.8)	0.001	1.1 (0.9–1.4)	0.326
SOFA	1.2 (1.2–1.3)	<0.001	1.2 (1.1–1.3)	<0.001

Adjusting variables were: age, septic shock, cardiovascular disease, immunosuppression, chronic renal failure, neoplasia, respiratory source of infection, renal replacement therapy, hospital (Valladolid/Dijon), presence of fungal infection, limitation of therapeutic effort.

### The influence of disease severity on biomarker performance

**Fig. 3:**
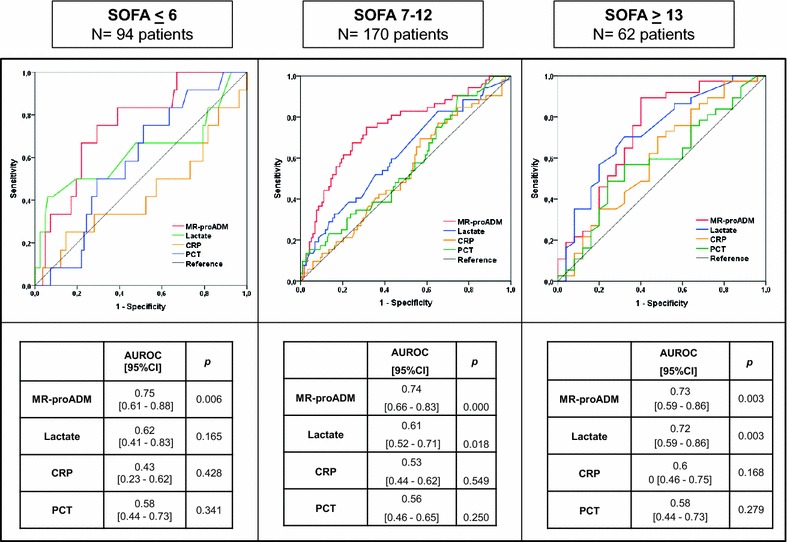
AUROC analysis for identifying non-survivors at 28 days depending on biomarker levels in the three severity groups

MR-proADM showed the best AUROC for mortality prediction at 28 days in the analysis over the entire cohort, even better than that of SOFA score (Fig. 2). When patients were stratified by the degree of organ failure, MR-proADM was the only biomarker able to discriminate non-survivors from survivors at 28 days in those patients with the lowest degree of disease severity (SOFA score ≤ 6), (AUROC [95% confidence interval (95% CI)] 0.75 [0.61–0.88]), (*p* = 0.006) (Fig. 3). In the moderately severe patients (SOFA score 7–12), MR-proADM showed a higher AUROC than that observed with lactate (0.74 [0.66–0.83] vs. 0.61 [0.52–0.71], respectively, Fig. 3). In the most severe patients (SOFA score ≥13), MR-proADM and lactate had a similar AUROC (0.73 [0.59–0.86] vs. 0.72 [0.59–0.86], respectively, Fig. 3). Neither CRP nor PCT was predictive of 28-day mortality in any severity group based on the SOFA score, with AUROCs ranging from 0.43 to 0.60.

The threshold values (cut-off) of MR-proADM were determined by having the highest specificity with a pre-fixed sensitivity of at least 0.80 for identifying non-survivors. For patients with SOFA scores ≤6, 7–12, and ≥13, the MR-proADM cut-offs were 1.79, 3.25, and 5.58 nmol/L, respectively (Table 3).

**Table 3:** MR-proADM cut-off (nmol/L) with the highest accuracy for predicting 28-day mortality based on SOFA score

	Cut-off	Sensitivity	Specificity	PPV	NPV	+LR	−LR
SOFA < 6	1.79	83.0	61.0	23.8	96.2	2.14	0.27
SOFA 7–12	3.25	83.0	52.0	43.4	87.0	1.74	0.33
SOFA ≥ 13	5.58	83.8	60.0	75.6	71.4	2.09	0.27

*PPV* positive predictive value, *NPV* negative predictive value, *+LR* positive likelihood ratio, *−LR* negative likelihood ratio

The length of ICU stay in each severity group was [mean, (SD)]: SOFA ≤ 6: 11.0 days (18.3); SOFA 7–12: 12.4 days (16.0) and SOFA ≥ 13: 8.4 days (7.6). The mortality rates for each severity group were 12.8, 30.6 and 59.7%, respectively.

### MR-proADM improves mortality prediction in the less severely ill patients

We evaluated the combination of MR-proADM and SOFA score in predicting mortality, such that patients with MR-proADM concentrations >1.79 nmol/L were considered to have a 1 point increase in the SOFA score. In patients with SOFA ≤ 6, the MR-proADM modified SOFA score (ADM-SOFA) showed an increased ability to identify non-survivors compared to SOFA alone, AUROC [95% CI] SOFA 0.70 [0.58–0.82] and ADM-SOFA 0.77 [0.66–0.88].

## Discussion

Severity in sepsis depends on the extent of organ failure as evaluated by the SOFA score, which in turn is directly associated with the risk of mortality [15]. Nonetheless, the emergence of an increasing number of biomarkers may provide a new avenue with which to improve prognostic accuracy in a simple and rapid manner. In this regard, our study suggests that MR-proADM may be a promising biomarker. However, previous studies evaluating the prognostic role of MR-proADM in sepsis have provided conflicting results. Christ-Crain et al. [16] found that MR-proADM yielded an AUROC of 0.81 for detecting ICU mortality in a group of 53 patients with sepsis. In contrast, Suberviola et al. [17] found limited value of MR-proADM for predicting hospital mortality in 137 sepsis patients, with an AUROC of 0.62. Yet Marino et al. [18] showed that in 101 patients with sepsis, severe sepsis or septic shock, plasma adrenomedullin was strongly associated with the severity of disease, vasopressor requirement and 28-day mortality. These divergent results on the prognostic role of MR-proADM may be explained by differences in patient characteristics, disease severity, infectious source, surgical versus medical and small sample sizes across the various studies.

In the present study, we demonstrated for the first time that the performance of biomarkers to predict mortality in sepsis strongly depends on the degree of organ failure upon ICU admission. Stratifying patients based on their SOFA score allowed us to demonstrate that MR-proADM was the only biomarker able to identify non-survivors in all the severity groups. This is particularly important for the less severely ill patients (SOFA score ≤ 6), since this group represents either the earliest presentation in the clinical course of sepsis and/or the less severe form of this disease.

Thus, MR-proADM may be a good candidate, after validation in further studies, to be incorporated in an early sepsis management protocol, since it can provide rapid prognostic value and help to guide diagnostic interventions and treatment decisions, consequently resembling the role of troponin in myocardial infarction or d-dimer in pulmonary embolism. The cut-off value of MR-proADM identified for this group of patients (1.79 nmol/L) could be very useful in this regard. This cut-off is able to detect mortality with a good sensitivity and a high negative predictive value. Thus, MR-proADM may potentially help stratify patients in clinical trials examining novel therapies for sepsis.

MR-proADM showed greater predictive value for the risk of mortality than other more commonly used biomarkers, including lactate, in patients with an intermediate degree of organ failure (SOFA score 7–12). In contrast, both MR-proADM and lactate performed similarly in the most severe patients (SOFA ≥ 13). Therefore, our results support the importance of considering the degree of organ failure when designing studies for the discovery of prognostic biomarkers in sepsis.

The assessment of organ failure by using the SOFA score was recently proposed by the SEPSIS-3 consensus to identify high risk patients with suspected infection [15]. Our results show that a “positive” MR-proADM value may improve the ability of SOFA to predict mortality in sepsis. Interestingly, a combination of MR-proADM with clinical scores such as PSI or CURB-65 also performed better than the clinical scores alone in patients with Community Acquired Pneumonia (CAP) or lower respiratory tract infections (LRTI) [12, 19–21]. As a result, MR-proADM could be used as a reliable risk-stratification tool with the ability to predict mortality or adverse events and to guide clinical decisions. Further clinical studies evaluating strategies combining MR-proADM with other classical severity scores and/or biomarkers for improving the recognition and prognostication of sepsis are therefore warranted [22, 23].

Finally, we observed that an MR-proADM value lower than 0.88 nmol/L may allow to “rule out” mortality in the 28 days following admission to the ICU. This cut-off may be especially useful for guiding early clinical decisions, when the clinical signs of overt organ failure are not yet apparent.

Indeed, our results are similar to those of previous studies. Albrich et al. found that patients with LRTI and MR-proADM concentrations <0.75 nmol/L had an overall mortality of less than 0.5% (11). Furthermore, Krüger et al. showed that patients with CAP and an MR-proADM concentration of <0.9 nmol/L had a survival probability of 99.3% (12). Bello et al. [24] also found an optimal MR-proADM cut-off for predicting 30-day mortality in patients with CAP of 1.06 nmol/L.

Our study is limited in that we evaluated MR-proADM and other biomarker levels only on the day of ICU admission. As a result, we cannot extrapolate our findings to the emergency department or general ward. MR-proADM monitoring over time may further illustrate a temporal trend, which can indicate the success of specific therapies and consequently increase its outcome predictive value [25]. Finally, in our cohort, MR-proADM levels slightly differed depending on the source of infection. Fungal infections induced the highest levels of MR-proADM, while viral infection induced the lowest. This was likely related to the fact that fungal infections resulted in a higher disease severity (median SOFA score of 12 vs. 9 points in patients with no fungal infection), while viral infections resulted in a milder disease severity (median SOFA score of 6.5 vs. 9 points in patients with no viral infection). The potential influence of the source of infection and the type of microbe on MR-proADM’s ability to predict mortality in sepsis merits further investigation.

## Conclusions

Our results demonstrate that the performance of biomarkers in determining the risk of mortality in sepsis is influenced by disease severity. In patients with moderate severity, MR-proADM outperformed other standard biomarkers. As a consequence, MR-proADM may aid the early identification of sepsis patients requiring urgent ICU admission as well as facilitating the subsequent clinical management of these patients.

## Additional file

**Additional file 1: Figure S1.** AUROC analysis for identifying non-survivors at 28 days based upon SOFA score.

## Abbreviations

ADM: adrenomedullin
MR-proADM: mid-regional proadrenomedullin
CRP: C-reactive protein
PCT: procalcitonin
SOFA: Sequential Organ Failure Assessment Score
CI: confidence interval
AUROC: area under receiver operating characteristics
PPV: positive predictive value
NPV: negative predictive value
LR: likelihood ratio
HR: hazard ratio
PSI: Pneumonia Severity Index
RRT: renal replacement therapy
