# Supplementary figures and images for: Superior accuracy of mid-regional proadrenomedullin for mortality prediction in sepsis with varying levels of illness severity
Source: Ann Intensive Care. 2017 Feb 10;7:15. doi: 10.1186/s13613-017-0238-9 (PMC5307393; doi:10.1186/s13613-017-0238-9)

## Slide 1
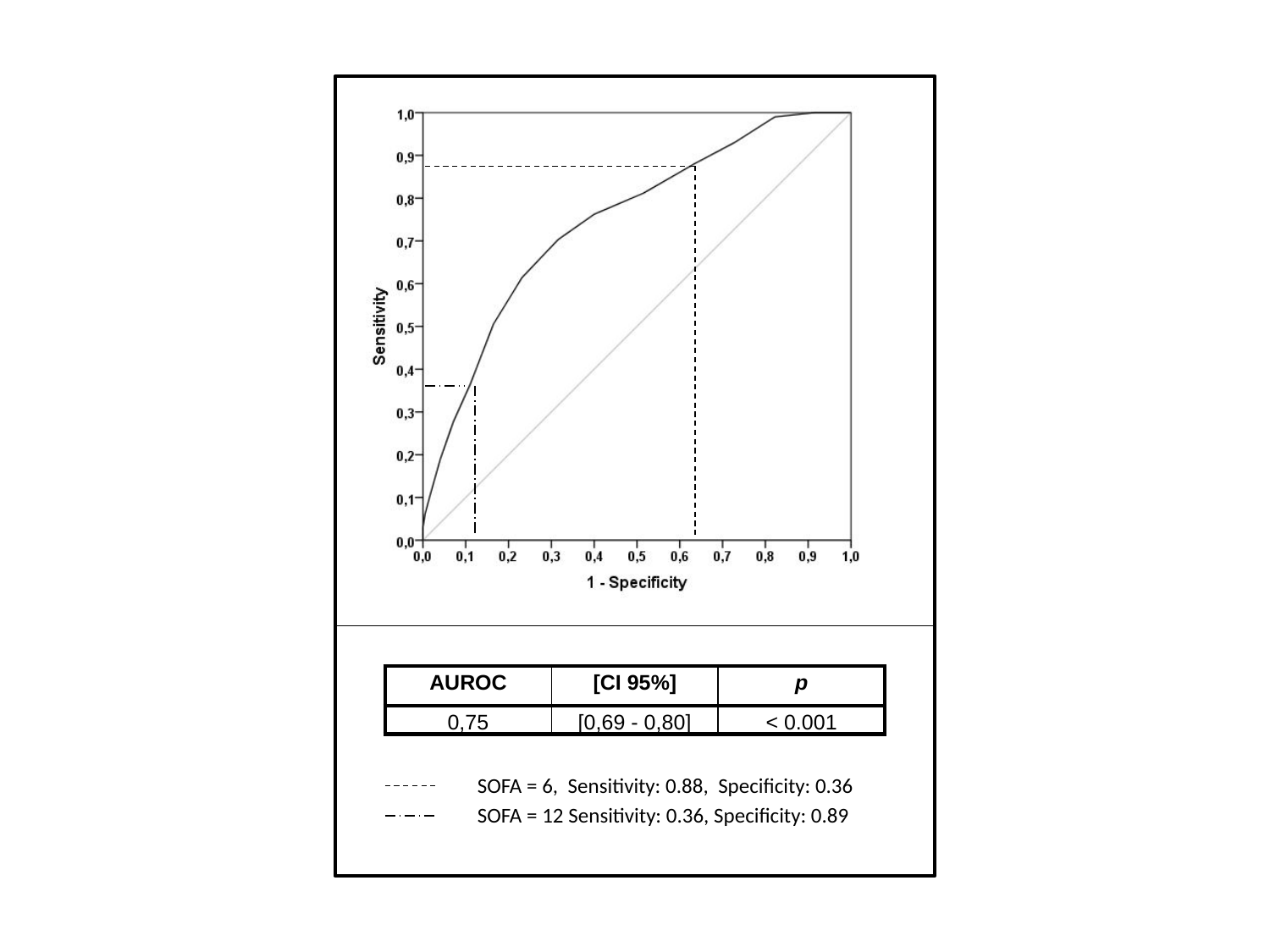

| AUROC | [CI 95%] | p |
| --- | --- | --- |
| 0,75 | [0,69 - 0,80] | < 0.001 |
SOFA = 6, Sensitivity: 0.88, Specificity: 0.36
SOFA = 12 Sensitivity: 0.36, Specificity: 0.89

Supplement: Supplementary file 1 — Additional file 1: Figure S1. AUROC analysis for identifying non-survivors at 28 days based upon SOFA score. [file 13613_2017_238_MOESM1_ESM.pptx]
